# Handling Missing Data in COVID-19 Incidence Estimation: Secondary Data Analysis

**DOI:** 10.2196/53719

**Published:** 2024-08-20

**Authors:** Hai-Thanh Pham, Toan Do, Jonggyu Baek, Cong-Khanh Nguyen, Quang-Thai Pham, Hoa L Nguyen, Robert Goldberg, Quang Loc Pham, Le Minh Giang

**Affiliations:** 1 School of Preventive Medicine and Public Health, Hanoi Medical University, 1 Ton That Tung Street, Kim Lien Ward, Dong Da District, Hanoi, 100000, Vietnam, 84 368-577-4236; 2 UMass Chan Medical School, University of Massachusetts Medical School, Worcester, MA, United States; 3 National Institute of Hygiene and Epidemiology, Hanoi, Vietnam

**Keywords:** imputation method, COVID-19 incidence rate, crude bias, crude RMSE, root mean square error, percentage change, pandemic, Vietnam, surveillance, population health, analytical method

## Abstract

**Background:**

The COVID-19 pandemic has revealed significant challenges in disease forecasting and in developing a public health response, emphasizing the need to manage missing data from various sources in making accurate forecasts.

**Objective:**

We aimed to show how handling missing data can affect estimates of the COVID-19 incidence rate (CIR) in different pandemic situations.

**Methods:**

This study used data from the COVID-19/SARS-CoV-2 surveillance system at the National Institute of Hygiene and Epidemiology, Vietnam. We separated the available data set into 3 distinct periods: zero COVID-19, transition, and new normal. We randomly removed 5% to 30% of data that were missing completely at random, with a break of 5% at each time point in the variable daily caseload of COVID-19. We selected 7 analytical methods to assess the effects of handling missing data and calculated statistical and epidemiological indices to measure the effectiveness of each method.

**Results:**

Our study examined missing data imputation performance across 3 study time periods: zero COVID-19 (n=3149), transition (n=1290), and new normal (n=9288). Imputation analyses showed that K-nearest neighbor (KNN) had the lowest mean absolute percentage change (APC) in CIR across the range (5% to 30%) of missing data. For instance, with 15% missing data, KNN resulted in 10.6%, 10.6%, and 9.7% average bias across the zero COVID-19, transition, and new normal periods, compared to 39.9%, 51.9%, and 289.7% with the maximum likelihood method. The autoregressive integrated moving average model showed the greatest mean APC in the mean number of confirmed cases of COVID-19 during each COVID-19 containment cycle (CCC) when we imputed the missing data in the zero COVID-19 period, rising from 226.3% at the 5% missing level to 6955.7% at the 30% missing level. Imputing missing data with median imputation methods had the lowest bias in the average number of confirmed cases in each CCC at all levels of missing data. In detail, in the 20% missing scenario, while median imputation had an average bias of 16.3% for confirmed cases in each CCC, which was lower than the KNN figure, maximum likelihood imputation showed a bias on average of 92.4% for confirmed cases in each CCC, which was the highest figure. During the new normal period in the 25% and 30% missing data scenarios, KNN imputation had average biases for CIR and confirmed cases in each CCC ranging from 21% to 32% for both, while maximum likelihood and moving average imputation showed biases on average above 250% for both CIR and confirmed cases in each CCC.

**Conclusions:**

Our study emphasizes the importance of understanding that the specific imputation method used by investigators should be tailored to the specific epidemiological context and data collection environment to ensure reliable estimates of the CIR.

## Introduction

Surveillance data are vital for public health policy and resource allocation [1]. During the COVID-19 pandemic, the rapid analysis of incomplete data led to potential biases, affecting our understanding of COVID-19 knowledge, attitudes, and behaviors [2]. Additionally, a study using US infectious disease surveillance data demonstrated that missing data can impact measured health disparities, emphasizing the need to consider this limitation when interpreting disparity metrics [3].

The absence of standardized and systematically collected surveillance data during the COVID-19 outbreak has necessitated the use of robust statistical tools and approaches to address these data gaps. Despite the availability of various analytical techniques, the application of statistical modeling processes has been limited [4]. Moreover, when imputation methods have been used, they have often lacked detailed descriptions and transparency [5].

Addressing the problem of missing data in public health surveillence systems requires system-level solutions, such as collecting more complete laboratory data, improving data linkage, and designing more efficient data collection procedures [3]. The analytical challenges posed by the current pandemic present an important opportunity to assess the utility of available statistical methods. Regardless of data quality, missing data and suboptimal analytical strategies can reduce a study’s statistical power and lead to biased estimates, resulting in erroneous conclusions. Robust statistical methods are crucial to enhance future data collection efforts, data interpretation, and their clinical and public health implications [6,7].

Gaps in the existing literature lie in the inadequate use of statistical modeling approaches to address the problem of missing data in disease and risk factor monitoring systems, particularly during public health emergencies such as the COVID-19 pandemic [8]. This shortfall is critical because missing data can significantly hinder the accurate monitoring of disease trends and the formulation of effective public health policies [9]. While various imputation methods exist, their application in this context has been limited, leading to uncertainties in disease trend forecasting and policy recommendations. These limitations can result in skewed data interpretations, which may, in turn, affect resource allocation, emergency response strategies, and overall public health outcomes.

In the present study, we used several theoretical approaches based on statistical modeling and epidemiological concepts to address the challenge of using different statistical methods for handling missing data in the interpretation of community surveillance information collected during different pandemic periods. We evaluated the performance of several imputation strategies to determine the best approaches for dealing with missing data in disease monitoring, showing how handling missing data can affect estimates of the COVID-19 incidence rate (CIR) in different pandemic situations.

## Methods

### Study Context and Data Source

This study used data collected in Bac Ninh Province, Vietnam, during the calendar year 2021 from the surveillance system for patients with COVID-19/SARS-CoV-2 who were admitted to the National Institute of Hygiene and Epidemiology in Hanoi, Vietnam.

The database included information on 13,727 patients with COVID-19 collected from the beginning of the 2021 outbreak in Bac Ninh Province, from January 1, 2021, to December 31, 2021, without any missing data. Based on the information contained in this data set, and because we wanted to restrict our study population to cases that could be transmitted to the broader community, we calculated the CIR only for confirmed cases of COVID-19 (n=10,599; this represents 77% of the data set) that were diagnosed in each community from each district in Bac Ninh Province (Multimedia Appendix 1).

We decided to focus exclusively on community cases to understand the transmission dynamics in the broader community. We focused on 3 specific variables in the data set: the date of each community-acquired case of COVID-19 that was forwarded to the surveillance system, the community code, and the number of daily cases at the community level (Multimedia Appendix 2).

### Overview

We conducted a simulation to calculate various statistical and epidemiological indices of this community epidemic, assessing the effectiveness of different methods for handling missing data across differing missingness proportions and pandemic periods for each of the 7 missing-data analytic methods. The simulation steps began with generating a reference data set by separating the data set into different periods. Subsequently, for each missingness proportion in each period, steps 2 through 4 were repeated, during which statistical and epidemiological indices were calculated for the 7 missing-data handling methods.

### Step 1: Separating by Period

We separated the COVID-19 pandemic that was occurring in Bac Ninh Province into 3 distinct time periods using the following working definitions: the first period, the zero COVID-19 period, ran from January 1 to July 4, 2021. This was when the local government had tightened prevention policies and the primary goal was to stop the community transmission of COVID-19. During this period, there were multiple short-range waves of COVID-19 outbreaks, with the peak CIR ranging from 150 to 250 cases daily [10].

The next period, the transition period, took place between July 5 and October 22, 2021. During this period, the local government used a flexible pandemic policy with the goal of controlling community transmission of COVID-19 and minimizing the importation of new cases from affected provinces while increasing the population level of COVID-19 vaccine coverage. During this period, the highest CIR was more than 200 cases per day, but there were many days in Bac Ninh province with no notification of cases (CIR=0), with the longest range of zero notification days being more than 2 weeks [10].

The final study period, the new normal, ran from October 23, 2021, until the end of the study on December 31, 2021. During this period, the primary goal of public health officials was to open social facilities and terminate all isolation policies. The CIR in this period fluctuated, with multiple long-range waves of outbreaks; during the highest peak, there were more than 600 daily cases of COVID-19 [10].

### Step 2: Generating Simulated Data Sets

We assumed that there were values missing completely at random in our study, so that the data values missing in our simulation data sets were unrelated to any observed or unobserved data in the data set. In other words, the missing data points did not depend on the values of other variables or the values of the missing variable itself. Inasmuch, we randomly changed the missing data percentage from 5% to 30%, with intervals of 5%, for each time point for the variable “cases per day at the community level.” This was defined as the total number of confirmed cases of COVID-19 that were diagnosed and reported daily at each community in Bac Ninh Province [8], resulting in 6 levels of cutoff percentages for missing data sets during each of the 3 distinct periods. We used the *missMethods* R package to generate missing values based on previous research that has shown the effectiveness of generating missing values in data sets [11]; 18 simulated databases were created in our study.

### Step 3: Handling the Missing Data

The methods for handling missing data were based on a previous literature review of the techniques used in ecological data sets [12]. We selected 7 methods that we deemed to be suitable for imputing missing values from the number of daily cases of COVID-19 occurring in each study community.

#### Backfill Imputation

We used the number of daily cases from the previous day for each community unit as the value for imputation for the missing values of that community. If there were no cases on the previous day to impute, we assumed a missing value of 0 because when no data were available from the previous day, assuming a value of 0 was a conservative approach, indicating no new cases reported. We used the “na. locf()” function in the *zoo* package of R to conduct this imputation process [13].

#### Moving Average

We used the mean of the last 14 days of COVID-19 as the average for imputation. The cutoff time of 14 days served as the reference for the minimum time for a COVID-19 containment cycle (CCC) [10]. We created a function to carry out this process.

#### Median Imputation

We created a function in R to use the value of the number of daily cases of COVID-19 during the last 14 days in each community as the reference to find the median for imputing missing values for that community.

#### Maximum Likelihood

We used maximum likelihood estimation (MLE), which is based on a normal distribution. We created a function to conduct this process. First, we calculated the MLE for the mean (μ) and SD (σ) of the last 14 days of nonmissing values in the input variable *x*. Then, for each missing value, we randomly sampled a value from a normal distribution with mean (μ) and SD (σ), effectively replacing the missing value.

#### Linear Interpolation

We use the “na_interpolation()” function in the *imputed* package of R [14]. Missing values were replaced by values estimated by linear interpolation, which created a linear relationship between neighboring known data points (the last day and the next day).

#### Autoregressive Integrated Moving Average Model

We used the “auto. arima()” function in the *forecast* package of R for calculating imputed missing values [15]. The autoregressive integrated moving average (ARIMA) model combines 3 key components: *AR* (the “autoregressive” term), *I* (the “differencing” term), and *MA* (the “moving average” term). The *AR* term refers to the past values used for forecasting the next value while the *MA* term is used to define the number of past forecast errors used to predict future values. The order of “differencing” specifies the number of times the differencing operation is performed on a series to make it stationary. In the default figures, the maximum number of historical observations was set to the last 5 days. The ARIMA model subsequently determined the order of these components (from 1 to 5 previous days might be possibly related to the current data), and imputation values were chosen through data analysis and model selection techniques.

#### K-Nearest Neighbor Imputation

We used the closest data points to the one with missing values. In our study, we used the “kNN()” function in the *VIM* package of R to fill in missing daily COVID-19 case counts at the community level by K-nearest neighbor (KNN) imputation [16]. This method estimates missing values based on nearby data points. We applied KNN with a set number of neighbors, in our example 14 days, representing the minimum time for a CCC in each community [10].

### Step 4: Estimating the Effectiveness

To illustrate the efficacy of various missing data handling methods in estimating the CIR, we implemented the 7 imputation techniques to address missing data during different study periods and levels of missing data.

On the statistical side, to assess the extent to which these missing data handling methods mitigated the effects on estimating the CIR, we examined bias and the root mean square error (RMSE) resulting from direct comparisons between the imputed and original values of the daily CIR for a population of 1,000,000 people. We computed the mean absolute crude bias ($\bar{ACB}$) and the mean crude RMSE ($\bar{RMSE}$) as indicators of performance [8]. To quantify the alterations in CIR between the original and the imputed data sets, we employed the mean absolute percentage change (APC) in the CIR, denoted as ${\bar{APC}}_{CIR}$ (Multimedia Appendix 3).

From the epidemiological perspective, we used the average number of confirmed cases in each CCC as the reference index to measure the effectiveness of the imputation data. The CCC consisted of several nonpharmacological control strategies aimed at managing the COVID-19 pandemic within each community in Bac Ninh Province [10]. We used the mean APC of the mean of the average confirmed cases of COVID-19 for each CCC, referred to as ${\bar{APC}}_{cases}$, to discern differences in confirmed cases for each CCC at the community level between the original and imputed data sets (Multimedia Appendix 3).

R (version 4.2.2; R Foundation for Statistical Computing) was used for all data analyses that were carried out.

### Ethical Considerations

The study received approval in accordance with decision 4326/QD-DHYHN by the Institutional Review Board of Hanoi Medical University. All methods were conducted in compliance with the committee’s guidelines and regulations. We received permission for all the data sets in this study from the Vietnam National Institute of Hygiene and Epidemiology for use and analysis. All personal information and identifiers were removed from the data set prior to analysis.

## Results

### Zero COVID-19 Period

**Figure 1.**
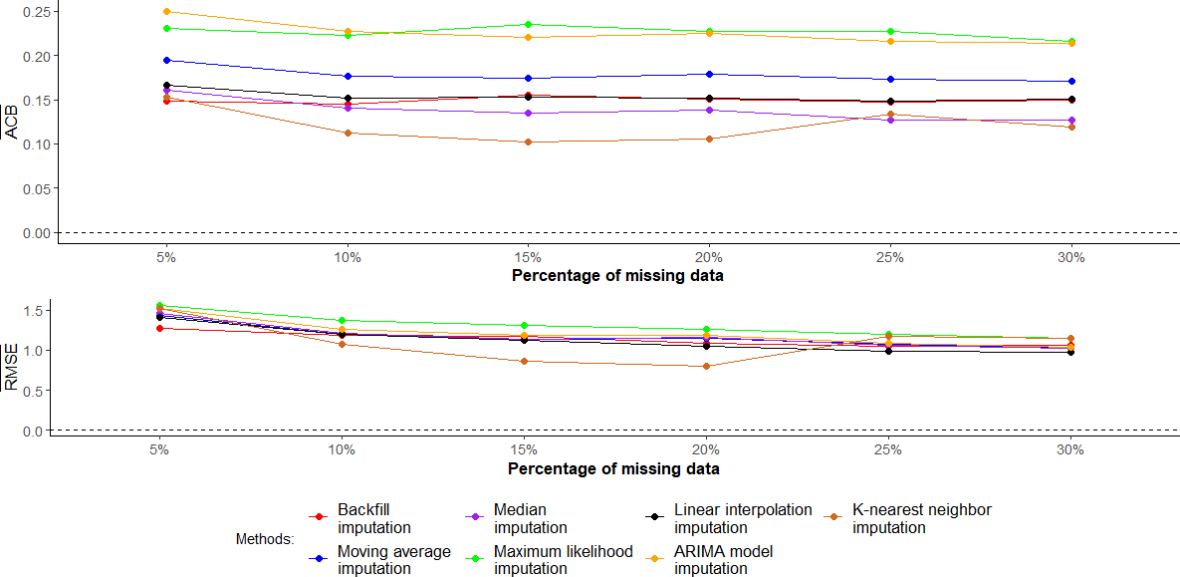
Mean absolute crude bias and mean crude root mean square error (RMSE) when using different imputation methods during the zero COVID-19 period. $\bar{ACB}$: the mean absolute crude bias of the COVID-19 incidence rate; ARIMA: autoregressive integrated moving average; $\bar{RMSE}$: mean crude RMSE of the COVID-19 incidence rate.

Figure 1 shows the results of the imputation methods used to address missing data in the context of the CIR during the zero COVID-19 period. Among these methods, KNN imputation showed the lowest mean ACB and mean crude RMSE values from 5% to 20% missing-data levels. In the 25% to 30% missing-data levels, while KNN imputation and median imputation consistently yielded lower mean ACBs than the other methods, linear interpolation imputation had the lowest mean crude RMSE.

Table 1 provides an assessment of the mean APC in the CIR and the average number of confirmed cases in each CCC during the zero COVID-19 period using the 7 imputation methods to address missing data. Median imputation and KNN imputation consistently exhibited the lowest mean APC values for both CIR and for the average number of confirmed cases during each CCC. The moving average imputation method followed as the second-lowest performer for APC in CIR, with the mean APC increasing gradually as the level of missing data increased. Backfill imputation was the second-lowest performer in APC in terms of the average number of confirmed cases during each CCC, with the mean APC value rising nearly similarly to the median imputation results. Both backfill imputation and median imputation had APCs on average for the number of confirmed cases of COVID-19 during each CCC higher than KNN imputation at all levels of missing data. In contrast, the linear interpolation imputation method consistently exhibited the highest mean APC values across the specified levels of missing data. Lastly, the ARIMA model imputation and maximum likelihood imputation methods demonstrated the second-highest mean APC values when missing data levels increased; the ARIMA model imputation had the highest APC on average for the number of confirmed cases of COVID-19 during each CCC.

**Table 1.** Mean absolute percentage change in the daily COVID-19 incidence rate (${\bar{APC}}_{CIR}$) and in the mean of the average of confirmed cases of COVID-19 during each COVID-19 containment cycle (${\bar{APC}}_{cases}$) when using different imputation methods during the zero COVID-19 period.

Imputation methods		Level of missing data, mean (SE)					
		5%	10%	15%	20%	25%	30%
**Backfill imputation**							
	${\bar{APC}}_{CIR}$	11.1 (8.2)	21.8 (11.1)	33.0 (13.3)	36.0 (13.3)	48.5 (19.4)	51.7 (19.6)
	${\bar{APC}}_{cases}$	4.5 (1.6)	13.1 (2.6)	19.6 (3.0)	28.9 (4.4)	35.6 (4.7)	44.3 (5.4)
**Moving average imputation**							
	${\bar{APC}}_{CIR}$	8.3 (2.0)	14.0 (2.4)	23.8 (3.9)	28.9 (4.8)	37.2 (6.5)	39.3 (6.6)
	${\bar{APC}}_{cases}$	24.8 (8.3)	57.6 (15.8)	77.1 (23.6)	215.3 (76.1)	244.2 (78.4)	269.0 (80.4)
**Median imputation**							
	${\bar{APC}}_{CIR}$	3.9 (1.0)	7.6 (1.4)	13.1 (2.2)	16.8 (2.6)	21.1 (3.1)	24.5 (3.2)
	${\bar{APC}}_{cases}$	4.3 (1.3)	14.3 (4.9)	18.6 (5.4)	64.5 (43.1)	36.0 (11.8)	42.5 (11.9)
**Maximum likelihood imputation**							
	${\bar{APC}}_{CIR}$	13.2 (4.2)	24.1 (7.2)	41.3 (10.7)	39.9 (8.0)	45.9 (7.6)	53.5 (11.0)
	${\bar{APC}}_{cases}$	24.8 (8.3)	57.6 (15.8)	77.1 (23.6)	215.3 (76.1)	244.2 (78.4)	269.0 (80.4)
**Linear interpolation imputation**							
	${\bar{APC}}_{CIR}$	15.5 (12.8)	26.9 (14.3)	33.6 (14.6)	39.5 (14.9)	48.8 (18.5)	56.2 (19.1)
	${\bar{APC}}_{cases}$	6.1 (2.2)	18.1 (3.8)	24.3 (4.0)	37.2 (6.1)	49.8 (6.6)	56.9 (7.0)
**Autoregressive integrated moving average model imputation**							
	${\bar{APC}}_{CIR}$	10.2 (1.4)	17.5 (2.4)	27.5 (3.7)	36.5 (4.9)	46.5 (6.4)	53.9 (7.7)
	${\bar{APC}}_{cases}$	226.3 (27.0)	544.4 (51.6)	1473.9 (238.7)	3295.6 (434.8)	5126.4 (551.1)	6955.7 (622.4)
**K-nearest neighbor imputation**							
	${\bar{APC}}_{CIR}$	3.7 (1.0)	6.9 (1.4)	10.6 (1.7)	10.3 (1.4)	15.8 (1.9)	17.8 (2.1)
	${\bar{APC}}_{cases}$	3.6 (0.7)	9.3 (1.7)	14.1 (2.1)	19.9 (2.7)	23.4 (3.4)	29.0 (3.2)

### Transition Period

**Figure 2.**
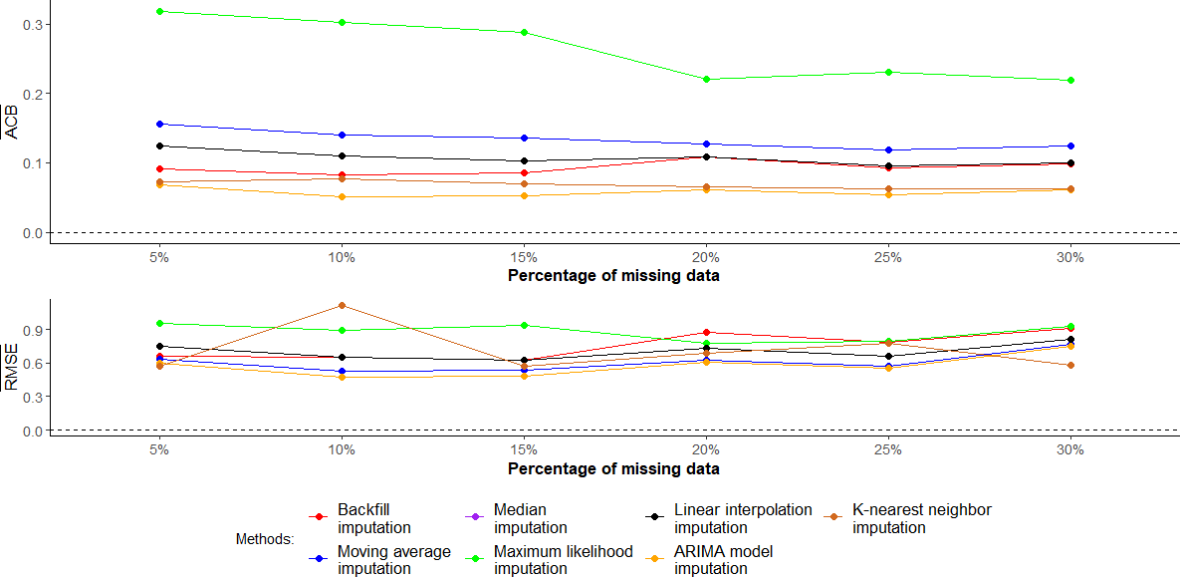
The mean absolute crude bias and mean crude root mean square error (RMSE) when using different imputation methods during the transition period. $\bar{ACB}$: the mean absolute crude bias of the COVID-19 incidence rate; ARIMA: autoregressive integrated moving average; $\bar{RMSE}$: mean crude RMSE of the COVID-19 incidence rate.

Figure 2 shows the results of the different imputation methods used to address missing data in the context of the CIR during the transition period. The ARIMA model and KNN imputation methods consistently demonstrated the lowest mean ACB across all levels of missing data; the ARIMA model and median imputation methods had the same results in terms of the mean ACB and mean RMSE. With regards to the mean crude RMSE, the moving average and ARIMA model imputation methods consistently yielded lower values than the other methods across varying levels of missing data. On the other hand, the maximum likelihood imputation method generally resulted in higher mean ACBs and mean crude RMSEs compared with alternative methods. The backfill imputation method exhibited the second-highest mean crude RMSE, particularly at the 20% to 30% level of missing data.

Table 2 presents an overview of the 7 imputation methods used to address missing data in the CIR and the average number of confirmed cases during each CCC. The median and ARIMA model imputation methods consistently displayed relatively lower mean APC values for both the CIR and average of confirmed cases in each CCC than the other analytic methods. The backfill imputation and KNN imputation methods provided the second-lowest mean APC values as the level of missing data increased. In contrast, the maximum likelihood and moving average imputation methods displayed comparatively higher mean APC values than the other methods of imputation.

**Table 2.** Mean absolute percentage change in the daily COVID-19 incidence rate (${\bar{APC}}_{CIR}$) and in the mean of the average number of confirmed cases of COVID-19 during each COVID-19 containment cycle (${\bar{APC}}_{cases}$) when using different imputation methods during the transition period.

Imputation methods		Level of missing data, mean (SE)					
		5%	10%	15%	20%	25%	30%
**Backfill imputation**							
	${\bar{APC}}_{CIR}$	2.8 (1.1)	12.3 (6.6)	15.9 (6.7)	16.7 (6.7)	19.1 (6.8)	26.0 (8.6)
	${\bar{APC}}_{cases}$	8.7 (2.4)	16.7 (3.3)	24.6 (4.0)	31.6 (4.4)	40.7 (5.3)	48.9 (5.3)
**Moving average imputation**							
	${\bar{APC}}_{CIR}$	11.4 (2.4)	20.0 (3.8)	30.4 (5.7)	31.4 (5.6)	34.9 (6.3)	42.7 (7.7)
	${\bar{APC}}_{cases}$	30.1 (3.4)	33.7 (4.4)	49.7 (11.4)	98.2 (17.8)	118.7 (19.0)	167.0 (25.3)
**Median imputation**							
	${\bar{APC}}_{CIR}$	3.0 (1.3)	3.6 (1.3)	6.6 (1.9)	8.7 (2.1)	10.6 (2.3)	12.43 (2.43)
	${\bar{APC}}_{cases}$	3.8 (1.4)	9.4 (2.6)	13.5 (3.0)	16.3 (3.2)	22.8 (3.8)	25.7 (3.9)
**Maximum likelihood imputation**							
	${\bar{APC}}_{CIR}$	18.6 (3.9)	32.6 (9.7)	51.9 (11.1)	54.4 (11.6)	58.3 (14.0)	48.8 (11.4)
	${\bar{APC}}_{cases}$	31.6 (3.6)	37.5 (6.1)	57.3 (12.9)	92.4 (19.3)	134.0 (22.3)	158.4 (24.9)
**Linear interpolation imputation**							
	${\bar{APC}}_{CIR}$	8.4 (3.6)	16.4 (5.4)	22.4 (7.5)	24.0 (7.5)	28.4 (7.8)	32.0 (9.5)
	${\bar{APC}}_{cases}$	9.8 (2.0)	18.8 (2.8)	25.5 (3.3)	30.8 (3.6)	37.2 (4.2)	44.4 (4.4)
**Autoregressive integrated moving average model imputation**							
	${\bar{APC}}_{CIR}$	3.0 (1.3)	3.7 (1.3)	6.7 (1.9)	8.7 (2.1)	10.6 (2.3)	12.4 (2.4)
	${\bar{APC}}_{cases}$	3.8 (1.4)	9.4 (2.6)	13.5 (3.0)	16.3 (3.2)	22.8 (3.8)	25.7 (3.9)
**K-nearest neighbor imputation**							
	${\bar{APC}}_{CIR}$	5.9 (2.0)	5.4 (1.7)	10.6 (2.5)	9.7 (2.0)	17.7 (3.9)	16.9 (3.1)
	${\bar{APC}}_{cases}$	5.6 (1.9)	8.8 (2.5)	13.5 (2.8)	17.0 (3.3)	12.7 (2.8)	22.3 (3.5)

### New Normal Period

**Figure 3.**
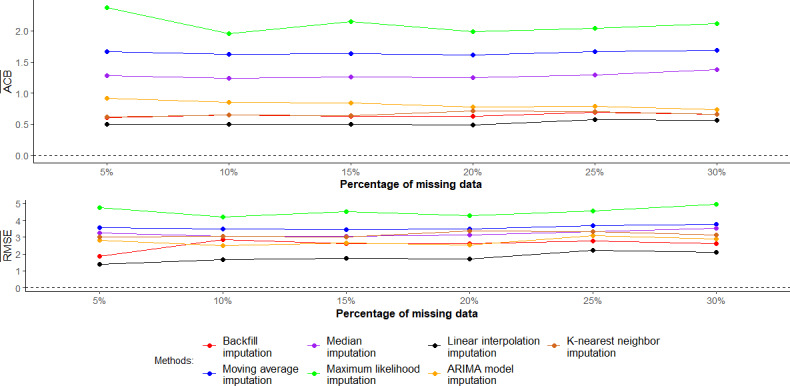
Mean absolute crude bias and mean crude root mean square error (RMSE) when using different imputation methods during the new normal period. $\bar{ACB}$: the mean absolute crude bias of the COVID-19 incidence rate; ARIMA: autoregressive integrated moving average; $\bar{RMSE}$: mean crude RMSE of the COVID-19 incidence rate.

Figure 3 illustrates the mean ACB and mean crude RMSE of the 7 imputation methods used to address missing data in the CIR during the new normal period. Both the backfill and linear interpolation imputation methods consistently demonstrated the lowest mean ACB across all levels of missing data. The ARIMA model imputation and KNN imputation methods provided the second-lowest absolute mean ACB and mean crude RMSE compared with the other analytic methods across different levels of missing data. On the other hand, the maximum likelihood and moving average imputation methods showed the highest mean ACB and mean crude RMSE as the level of missing data increased.

Table 3 displays the mean APC between the original and imputation data sets when we addressed varying levels of missing data in the CIR and in the average number of confirmed cases in each CCC during the new normal period. Three statistical methods, namely the backfill, linear interpolation, and KNN imputation methods, consistently exhibited relatively lower mean APC values compared with the other imputation methods. While the ARIMA model imputation method provided the second-lowest mean APC in CIR values as the level of missing data increased, median imputation had the second-lowest mean APC in terms of the average number of confirmed cases in each CCC at all levels of missing data. In contrast, the maximum likelihood and moving average imputation methods consistently displayed higher mean APC values in the CIR and in the average number of confirmed cases during each CCC than the other methods of imputing missing data.

**Table 3.** Mean absolute percentage change in the daily COVID-19 incidence rate (${\bar{APC}}_{CIR}\;$) and in the mean of the average number of confirmed cases of COVID-19 during each COVID-19 containment cycle (${\bar{APC}}_{cases}$) when using different imputation methods during the new normal period.

Imputation methods		Level of missing data, mean (SE)					
		5%	10%	15%	20%	25%	30%
**Backfill imputation**						
	${\bar{APC}}_{CIR}$	7.9 (5.7)	15.6 (8.1)	19.8 (9.6)	23.9 (10.1)	28.9 (10.2)	39.3 (12.5)
	${\bar{APC}}_{cases}$	8.3 (1.8)	14.1 (2.2)	21.5 (3.1)	29.9 (4.0)	38.36 (5.12)	45.84 (5.79)
**Moving average imputation**						
	${\bar{APC}}_{CIR}$	80.9 (30.0)	189.7 (79.2)	301.2 (117.2)	390.3 (144.4)	491.7 (194.2)	578.1 (212.3)
	${\bar{APC}}_{cases}$	54.6 (6.6)	102.2 (11.3)	142.7 (14.8)	220.7 (17.9)	259.7 (19.4)	303.2 (21.4)
**Median imputation**						
	${\bar{APC}}_{CIR}$	58.5 (25.0)	134.7 (65.0)	215.0 (89.8)	279.8 (111.6)	359.3 (152.0)	439.1 (171.7)
	${\bar{APC}}_{cases}$	25.5 (5.2)	45.4 (8.3)	66.2 (10.6)	104.3 (14.9)	124.2 (16.6)	158.1 (20.8)
**Maximum likelihood imputation**						
	${\bar{APC}}_{CIR}$	92.1 (33.8)	213.8 (89.7)	289.7 (107.0)	321.3 (141.7)	472.5 (187.5)	605.7 (235.9)
	${\bar{APC}}_{cases}$	58.6 (7.6)	105.5 (11.5)	145.0 (15.8)	221.8 (19.5)	262.5 (20.9)	313.7 (24.2)
**Linear interpolation imputation**						
	${\bar{APC}}_{CIR}$	4.8 (2.9)	8.6 (3.6)	10.9 (4.3)	13.4 (4.6)	29.6 (10.8)	35.7 (11.3)
	${\bar{APC}}_{cases}$	9.2 (2.4)	15.8 (3.0)	23.6 (3.7)	31.2 (4.3)	41.3 (5.4)	44.8 (5.6)
**Autoregressive integrated moving average model imputation**						
	${\bar{APC}}_{CIR}$	22.0 (7.0)	43.5 (15.3)	62.7 (21.2)	72.6 (24.0)	58.4 (17.7)	69.5 (20.9)
	${\bar{APC}}_{cases}$	50.5 (5.7)	87.9 (9.4)	118.4 (12.8)	174.5 (15.0)	169.3 (13.2)	190.1 (14.1)
**K-nearest neighbor imputation**						
	${\bar{APC}}_{CIR}$	4.5 (1.6)	8.5 (1.9)	9.7 (1.4)	11.3 (1.5)	23.0 (3.1)	21.2 (2.8)
	${\bar{APC}}_{cases}$	4.2 (0.8)	12.7 (2.3)	16.6 (1.8)	25.0 (2.4)	24.7 (2.2)	32.6 (2.7)

## Discussion

### Principal Results

In examining our study’s primary objective, which was to demonstrate how different methods of handling missing data affect estimation of the CIR, we highlight how the ongoing pandemic, as well as the preventive measures and health policy recommendations that were used to control future cases of COVID-19 in the community, could affect the effectiveness of different analytical methods. After examining 7 imputation approaches, we found that KNN and median imputation performed the best during the zero COVID-19 period, with KNN also having the lowest mean APC in terms of the CIR. ARIMA and median imputation were the most successful analytic approaches used during the transition period, whereas backfill, linear interpolation, and KNN performed the best during the new normal phase. Inasmuch, our findings show that one’s selection of the different imputation methods that could be used must take into account the specific pandemic conditions to increase the accuracy of predicted incidence rate estimates.

### Comparisons With Prior Work

Several of our findings differ from those of a study that was designed to find the best way to handle missing data for estimating a wellness index over the lifetime based on panel data from smart devices that collected various types of life logs, such as steps walked and sleep duration [17]. Our findings also differ from a study that examined how well artificial neural networks handle missing data collected in a pediatric intensive care unit [18]. The differences between our results and these previous studies were due to the performance of different imputation methods and a focus on different pandemic time periods. This underscores the importance of understanding the particular pandemic situation and developing and using health policy measures considering the potential biases and effectiveness of these analytic techniques. During periods of strict population-based control, such as the zero COVID-19 period, simpler methods, such as KNN and median imputation methods, which rely on recent data, could be used. In contrast, during more volatile periods of viral infections, such as the transition and new normal periods, methods that model temporal dependencies or use neighboring data points, namely the ARIMA model and KNN imputation methods, are more effective.

Our results also highlight the limitations of certain analytic methods, such as the maximum likelihood and moving average, which generally showed higher mean ACB and crude RMSE values, indicating less robustness in handling variability in the extent of missing data during different pandemic phases. These methods are, however, often used to handle missing data in medical data sets. For example, in a study involving 50 individuals selected from a 2 × 2 randomized controlled trial, the moving average method showed the best agreement with observed values [19]. This study compared various data imputation methods for calculating body weight variability using both linear and nonlinear approaches. Moreover, maximum likelihood imputation methods have been used for handing missing data at random in a number of randomized controlled trials [20]. The limitations of these methods in our study may be attributed to their underlying assumptions, which might not hold in the rapidly changing context of a pandemic, leading to increased bias and error in the calculation and interpretation of imputed data and illness incidence rates.

The effectiveness of each imputation method that we used in this study was influenced by the underlying data structure and characteristics of missingness during each pandemic period. For example, the backfill method, which assumes that the last observed value can be carried forward, may work during periods of low variability but can introduce significant bias during high variability periods, such as the new normal period. Similarly, moving average methods might not capture true variability in the number of cases of disease that may occur during rapid changes in transmission dynamics. Our findings differ from an observational study in 2023 that used moving average imputation for 3 public, completed time-series data sets that were collected from power equipment [21]. This study aimed to create a customized methodology that combined an asymmetric denoising autoencoder and a moving average filter to impute missing data in time-series monitoring data. When choosing different imputation methods, it is crucial to consider epidemic-specific and contextual factors. Data may be missing due to overwhelmed health care systems or reporting delays in the number of cases of confirmed illness, leading to errors in data interpretation and policy recommendations to contain the spread of disease. In addition, the stage of the epidemic and extent of use and effectiveness of public health interventions can impact the suitability of different imputation techniques. Understanding these factors is essential to selecting methods that minimize bias and accurately reflect underlying trends in disease magnitude and health-related outcomes.

These findings emphasize the need for transparency and detailed reporting in the application of data imputation methods. The lack of detailed descriptions and transparency in the reporting and application of these methods in previous studies has been a significant limitation in interpreting the published literature. By providing a comprehensive analysis of various imputation techniques and their performance across different pandemic phases, this study contributes to a better understanding of how to more effectively handle missing data in disease surveillance. The detailed comparison of methods and the consideration of different pandemic phases provide valuable insights for future research and public health practice.

### Study Strengths and Limitations

The main strength of this study is that we used individual data to calculate the number of new cases of COVID-19 that were diagnosed and reported to public health authorities on a daily basis in each of the communities studied. Moreover, we were able to compare the original values with the imputed estimates that were collected during the 3 periods of this ongoing epidemic in a large Vietnamese province.

There are some limitations of our study, however, that need to be kept in mind in the interpretation of our principal study findings. Because we targeted an extensive range of missing values greater than 5%, we did not use any methods to ignore missing data or delete the missing values, such as listwise deletion or pairwise deletion. In addition, we used large imputed data sets and did not use methods useful for handling missing data in studies with small sample sizes, such as data augmentation [22]. Furthermore, our results are primarily limited to handling missing data with missing completely at random patterns without a need to account for potential biases that may have been introduced by nonrandom missing data. Scenarios in which data were missing at random or missing not at random were not addressed in this study [23]. Future investigations will be needed to analyze these types of missing-data scenarios. Another limitation is that our study did not account for unexpected cases, as the data were produced based on existing data and therefore may not represent some unforeseen phenomena.

### Conclusions

This study illustrates that the choice of imputation method used should be tailored to the specific epidemiological context and data collection environment. Statistical modeling and a thorough understanding of local pandemic dynamics are essential for improving the accuracy of incidence rate estimates and, in turn, public health responses to ongoing disease trends and the development and application of disease control measures. Future research should continue to refine these methods, ensuring that they can adapt to the evolving challenges of disease surveillance in public health emergencies. By improving currently available imputation methods, we can facilitate more accurate and dependable public health responses in future situations, ultimately contributing to better resource allocation, emergency response strategies, and community health outcomes.

## Supplementary material

10.2196/53719 Multimedia Appendix 1 Study population characteristics according to the study period.

10.2196/53719 Multimedia Appendix 2 Example of data sets and characteristics of study variables.

10.2196/53719 Multimedia Appendix 3 Study formulas.

## Abbreviations

ACB: absolute crude bias
APC: absolute percentage change
ARIMA: autoregressive integrated moving average
CCC: COVID-19 containment cycle
CIR: COVID-19 incidence rate
KNN: K-nearest neighbor
MLE: maximum likelihood estimation
RMSE: root mean square error
