# Supplementary material for: Handling Missing Data in COVID-19 Incidence Estimation: Secondary Data Analysis
Source: JMIR Public Health Surveill. 2024 Aug 20;10:e53719. doi: 10.2196/53719 (PMC11350390; doi:10.2196/53719)
Supplement: Multimedia Appendix 1 [file publichealth-v10-e53719-s001.docx]

# Multimedia Appendix 1

##### Appendix Table 1. Study population characteristics according to study time period

| Characteristics  n (%) | Period | | | P |
| --- | --- | --- | --- | --- |
|  | Zero-COVID | Transition | New-normal |  |
|  | (n=3149) | (n=1290) | (n=9288) |  |
| Age (years) |  |  |  |  |
| Mean (SD) | 30.1 (16.1) | 29.2 (15.2) | 28.3 (13.9) | *P< .001 ^a^* |
| Age group |  |  |  |  |
| < 10 | 293 (9.3) | 128 (9.9) | 763 (8.2) | *P< .001* |
| 10 - 19 | 465 (14.8) | 205 (15.9) | 1266 (13.6) |  |
| 20 - 29 | 967 (30.7) | 385 (29.8) | 3662 (39.4) |  |
| 30 - 39 | 756 (24.0) | 297 (23.0) | 2156 (23.2) |  |
| 40 - 49 | 278 (8.8) | 153 (11.9) | 682 (7.3) |  |
| 50 - 59 | 204 (6.5) | 60 (4.7) | 417 (4.5) |  |
| >= 60 | 186 (5.9) | 62 (4.8) | 341 (3.7) |  |
| Sex |  |  |  |  |
| Male | 1438 (45.7) | 567 (44.0) | 4648 (50.1) | *P< .001* |
| Female | 1711 (54.3) | 723 (56.0) | 4640 (49.9) |  |
| Living place |  |  |  |  |
| Bac Ninh City | 1186 (37.7) | 351 (27.2) | 2749 (29.6) | *P< .001* |
| Yen Phong District | 132 (4.2) | 183 (14.2) | 552 (5.9) |  |
| Que Vo District | 410 (13.0) | 192 (14.9) | 1531 (16.5) |  |
| Tien Du District | 109 (3.5) | 79 (6.1) | 611 (6.6) |  |
| Tu Son Town | 293 (9.3) | 182 (14.1) | 1608 (17.3) |  |
| Thuan Thanh District | 712 (22.6) | 46 (3.6) | 449 (4.8) |  |
| Gia Binh District | 92 (2.9) | 10 (0.8) | 261 (2.8) |  |
| Luong Tai District | 28 (0.9) | 104 (8.1) | 204 (2.2) |  |
| Other Provinces | 187 (5.9) | 143 (11.1) | 1323 (14.2) |  |
| Case classification |  |  |  |  |
| Community areas | 2674 (84.9) | 861 (66.7) | 7064 (76.1) | *P< .001* |
| Quarantine areas | 475 (15.1) | 429 (33.3) | 2224 (23.9) |  |

^a^ Multiple pairwise-comparison: Zero-COVID vs Transition: *P = .20*; Transition vs New-normal: *P = .06*; Zero-COVID vs New-normal: *P < .001*
