# Supplementary material for: Handling Missing Data in COVID-19 Incidence Estimation: Secondary Data Analysis
Source: JMIR Public Health Surveill. 2024 Aug 20;10:e53719. doi: 10.2196/53719 (PMC11350390; doi:10.2196/53719)
Supplement: Multimedia Appendix 2 [file publichealth-v10-e53719-s002.docx]

# Multimedia Appendix 2

##### Appendix Table 2. Example of datasets

| ID | community_code | report_day | case | period |
| --- | --- | --- | --- | --- |
| 1 | 09163 | 2021-05-17 | 2 | Zero - COVID |
| 2 | 09163 | 2021-05-21 | 4 | Zero - COVID |
| 3 | 09163 | 2021-05-23 | 1 | Zero - COVID |
| 4 | 09163 | 2021-05-28 | 2 | Zero - COVID |
| 5 | 09163 | 2021-05-31 | 1 | Zero - COVID |
| 6 | 09163 | 2021-06-02 | 2 | Zero - COVID |
| 7 | 09163 | 2021-06-03 | 2 | Zero - COVID |
| 8 | 09163 | 2021-08-12 | 1 | Transition |
| 9 | 09163 | 2021-10-12 | 1 | Transition |
| 10 | 09163 | 2021-10-29 | 1 | New-normal |
| 11 | 09163 | 2021-10-30 | 1 | New-normal |
| 12 | 09163 | 2021-11-11 | 1 | New-normal |
| 13 | 09163 | 2021-11-13 | 1 | New-normal |
| 14 | 09163 | 2021-11-23 | 1 | New-normal |
| 15 | 09163 | 2021-11-25 | 2 | New-normal |
| 16 | 09163 | 2021-11-26 | 1 | New-normal |
| 17 | 09163 | 2021-12-11 | 1 | New-normal |
| 18 | 09163 | 2021-12-13 | 1 | New-normal |
| 19 | 09163 | 2021-12-15 | 1 | New-normal |
| 20 | 09163 | 2021-12-16 | 1 | New-normal |

^a^ Variable definition: “community_code”: Community code; “report_day”: Report Date; “case”: Case per day at the community level; “period”: Study period.

##### Appendix Table 3. Characteristics of study variables

| I.D. | Name of variable | Definition | Type of variable |
| --- | --- | --- | --- |
| 1 | Community code | Living community code of study subject | Character |
| 2 | Case per day at the community level | The total number of communities confirm cases per day at each community in Bac Ninh Province. | Count |
| 3 | Report Date | Reporting date of when identified communities confirm cases at the community level | Date |
