# Supplementary material for: Handling Missing Data in COVID-19 Incidence Estimation: Secondary Data Analysis
Source: JMIR Public Health Surveill. 2024 Aug 20;10:e53719. doi: 10.2196/53719 (PMC11350390; doi:10.2196/53719)
Supplement: Multimedia Appendix 3 [file publichealth-v10-e53719-s003.docx]

# Multimedia Appendix 3

## The Mean Absolute Crude Bias ($\bar{\boldsymbol{ACB}}$) *formula*

| $CIR= \frac{x}{P}$ * 1,000,000 | (1) |
| --- | --- |
| $\mathrm{ACB}_{c}= \frac{\sum_{i=1}^{M_{c}} \left\vert\mathrm{CIR}_{c_{i}}^{\mathrm{Imputation}}-\mathrm{CIR}_{c_{i}}^{\mathrm{Original}} \right\vert}{M_{c}}$ | (2) |
| $\bar{\mathrm{ACB}}= \frac{\sum_{j=1}^{N_{c}} \mathrm{ACB}_{c_{j}}}{N_{c}}$ | (3) |
|  |  |

In the formula:

- CIR was the daily COVID-19 incidence rate in 1,000,000 residents of Bac Ninh Province.
- P was the population of Bac Ninh Province (which was 1,368,840 [23]).
- x was the number of confirmed cases of COVID-19 diagnosed daily in Bac Ninh Province.
- $\mathrm{AC}B_{c}$ was the absolute crude bias in the community $c$ that had missing data during the study period.
- $\bar{\mathrm{ACB}}$ was the overall mean of absolute crude bias across communities during the study period.
- x was the number of confirmed cases of COVID-19 observed in Bac Ninh Province daily.
- i was the day of the study period.
- M_c_ was the number of days that had missing value in each community during the study period.
- N_c_ was the number of communities that had missing values during the study period.
- j was the number of communities that had confirmed cases of COVID-19 at day i during the study period.

## The Mean crude RMSE ($\bar{\boldsymbol{RMSE}}$) formula

| $\mathrm{RMSE}_{c}= \sqrt{\frac{{\sum_{i=1}^{M_{c}} \left( \mathrm{CIR}_{c_{i}}^{\mathrm{Imputation}}-\mathrm{CIR}_{c_{i}}^{\mathrm{Original}} \right)^{2}}}{M_{c}}}$ | (4) |
| --- | --- |
| $\bar{\mathrm{RMSE}}= \frac{\sum_{j=1}^{N_{c}} \mathrm{RMSE}_{c_{j}}}{N_{c}}$ | (5) |

In the formula:

- CIR was the daily COVID-19 incidence rate in 1,000,000 residents of Bac Ninh Province.
- $\mathrm{RMS}E_{c}$ was the crude RMSE from the community $c$ that had missing data during the study period.
- $\bar{\mathrm{RMSE}}$ was the overall mean of crude RMSE across communities during the study period.
- i was the day of the study period.
- M_c_ was the number of days with missing values in each community during the study period.
- N_c_ was the number of communities that had missing values during the study period.
- j was the number of communities that had confirmed cases of COVID-19 at day i during the study period.

## The Mean Absolute Percentage Change in the CIR ( ${\bar{\boldsymbol{APC}}}_{\boldsymbol{CIR}}$ ) formula

| $\mathrm{APC}_{\mathrm{CIR}}= \frac{\sum_{i=1}^{D} \left\vert\mathrm{CIR}_{i}^{\mathrm{Imputation}}-\mathrm{CIR}_{i}^{\mathrm{Original}} \right\vert}{\mathrm{CIR}_{i}^{\mathrm{Original}}}$ * 100 | (6) |
| --- | --- |
| ${\bar{\mathrm{APC}}}_{\mathrm{CIR}}= \frac{\sum_{i=1}^{D} \mathrm{APC}_{\mathrm{CIR}}}{D}$ | (7) |

In the formula:

- $\mathrm{CIR}_{i}$ was the daily COVID-19 incidence rate in 1,000,000 residents of Bac Ninh Province on the day i during the study period.
- $\mathrm{APC}_{\mathrm{CIR}}$ was the absolute percentage change in the $\mathrm{CIR}_{i}$ at day i during the study period.
- ${\bar{\mathrm{APC}}}_{\mathrm{CIR}}$ was the mean absolute percentage change in the CIR during the study period.
- D was the number of days during the study period.

## The Mean Absolute Percentage Change of the mean of the average confirmed cases of COVID-19 for each CCC cycle, referred to as (${\bar{\boldsymbol{APC}}}_{\boldsymbol{cases}}$) formula

| $\mathrm{ACC}_{c}= \frac{\sum_{j=1}^{T_{c}} y_{c_{j}}}{z_{c_{j}}}$ | (8) |
| --- | --- |
| $\bar{\mathrm{ACC}}= \frac{\sum_{j=1}^{T_{c}} \mathrm{ACC}_{c_{j}}}{T_{c}}$ | (9) |
| ${\mathrm{APC}_{\mathrm{cases}}}_{c}= \frac{\sum_{j=1}^{T_{c}} \left\vert{\bar{\mathrm{ACC}}}_{c_{j}}^{\mathrm{Impute}}- {\bar{\mathrm{ACC}}}_{c_{j}}^{\mathrm{Original}} \right\vert}{{\bar{\mathrm{ACC}}}_{c_{j}}^{\mathrm{Original}}}$ * 100 | (10) |
| ${\bar{\mathrm{APC}}}_{\mathrm{cases}}= \frac{\sum_{j=1}^{T_{c}} {{\mathrm{APC}_{\mathrm{cases}}}_{c}}_{j}}{T_{c}}$ | (11) |

In the formula:

- $\mathrm{ACC}_{c}$ was the average of confirmed cases of each CCC cycle in each community.
- $\bar{\mathrm{ACC}}$ was the mean of the average of confirmed cases of each CCC cycle in each community.
- $\mathrm{APC}_{\mathrm{cases}}$was the absolute percentage change of the mean of the average of confirmed cases of each CCC cycle at each community during the study period.
- ${\bar{\mathrm{APC}}}_{\mathrm{cases}}$ was the mean absolute percentage change of the mean of the average of confirmed cases of each CCC cycle at each community during the study period.
- $y_{c}$ was the number of confirmed cases of each CCC cycle in each community during the study period.
- $z_{c}$ was the total number of CCC cycles in each community during the study period.

T_c_ was the total of community-confirmed cases during the study period.
